# Ethics and regulation of inter-country medically assisted reproduction: a call for action

**DOI:** 10.1186/s13584-016-0117-0

**Published:** 2016-12-07

**Authors:** Carmel Shalev, Adi Moreno, Hedva Eyal, Michal Leibel, Rhona Schuz, Talia Eldar-Geva

**Affiliations:** 1 Department for Reproduction and Society, International Center for Health Law and Ethics, Haifa University, Haifa, Israel; 2 Morgan Centre for Research, University of Manchester, Manchester, UK; 3 Federmann School of Public Policy and Government, Hebrew University of Jerusalem, Jerusalem, Israel; 4 Task Force on Human Trafficking, Jerusalem, Israel; 5 Center for the Rights of the Child and the Family, Sha’arei Mishpat Law School, Hod HaSharon, Israel; 6 Department of Gynecology, Shaare Zedek Medical Center, Hebrew University of Jerusalem, Jerusalem, Israel

**Keywords:** Medically assisted reproduction, Cross-border reproductive care, Reproductive ethics, Human trafficking, Surrogacy, Egg donation, Ethic of care, Human rights

## Abstract

The proliferation of medically assisted reproduction (MAR) for the treatment of infertility has brought benefit to many individuals around the world. But infertility and its treatment continue to be a cause of suffering, and over the past decade, there has been a steady growth in a new global market of inter-country medically assisted reproduction (IMAR) involving ‘third-party’ individuals acting as surrogate mothers and gamete donors in reproductive collaborations for the benefit of other individuals and couples who wish to have children. At the same time there is evidence of a double standard of care for third-party women involved in IMAR, violations of human rights of children and women, and extreme abuses that are tantamount to reproductive trafficking. This paper is the report of an inter-disciplinary working group of experts who convened in Israel to discuss the complex issues of IMAR. In Israel too IMAR practices have grown rapidly in recent years, mainly because of restrictions on access to domestic surrogacy for same sex couples and a chronically insufficient supply of egg cells for the treatment of couples and singles in need. Drawing upon local expertise, the paper describes documented practices that are harmful, suggests principles of good practice based on an ethic of care, and calls for action at the international, national and professional levels to establish a human rights based system of international governance for IMAR based on three regulatory models: public health monitoring, inter-country adoption, and trafficking in human beings, organs and tissues.

**Electronic supplementary material:**

The online version of this article (doi:10.1186/s13584-016-0117-0) contains supplementary material, which is available to authorized users.

## Preface: an emerging global market

The proliferation of medically assisted reproduction (MAR) for the treatment of infertility has brought benefit to many individuals around the world, since the first birth of a child following in vitro fertilization (IVF) in 1978. By 2012 it was estimated that the number of babies born as a result of MAR reached a total of 5 million [1]. Infertility is often a cause of suffering and of social harm, particularly to women, and the right to reproductive health can be understood to include a right to treatment of infertility. But women also carry the primary burden of treatment for others: IVF is used for the treatment of male infertility; IVF also serves as a platform technology for pre-implantation genetic diagnosis (PGD) of embryos, often with no medical cause and as a tool for elimination of female fetuses. All these, together with the spread of egg ‘provision’^1^ practices and surrogacy arrangements, mean that more often than not otherwise healthy women undergo invasive medical interventions for the sake of their partners or for strangers who wish to become parents.

What is more, over the past decade, there has been a steady growth in a new global market of cross-border medical travel for repro-genetic purposes. Many practices of inter-country medically assisted reproduction (IMAR) involve ‘third-party’ individuals acting as surrogate mothers and gamete providers in reproductive collaborations for the benefit of other individuals and couples who wish to have children. IMAR involves various permutations of the cross-border movement of intended parents, third-party reproductive collaborators and new-born children, with transfers of human embryos, sperm and egg cells. Like transnational organ transplants, IMAR consists of shifting international networks. The chain of medical production starts from sperm and egg cell procurement, and continues through fertilization, embryo implantation and gestation, to culminate in birthing. Theoretically each of these six links could be performed in a different country, and the child then transported to the country of the intended parents. Some of the surrogacy practices currently marketed involve, in combination, three different provider countries. The intended parents from country A might transact with an egg provider from country B, who travels to a clinic in country C, where the egg is fertilised and implanted in a surrogate mother from provider country D (Eyal H, Samama E, Shalev C. Transnational surrogacy and the earthquake in Nepal: a case study from Israel. In: Miranda Davis [ed], Global Babies: Transnational Surrogacy and the New Politics of Reproduction (Zed Books, forthcoming 2017)).

The growth of the IMAR market in recent years is due to complex economic, legal and cultural conditions. A major driver of this multi-billion dollar business is the desire of individuals to parent children, and their inability to do so in their home countries due to legal restrictions or economic constraints on surrogacy or egg cell procurement. Moreover, there are signs of an emerging market of cross-border reproductive care for non-medical sex selection of embryos by means of PGD, and similar practices for the selection of preferred embryonic traits are likely to grow further [2]. Since the IMAR market is not regulated, there is no official data and a dearth of information. At the same time, for-profit trade in IMAR services involves the commodification of human beings (women and children) and body parts (gametes and wombs). Indeed, there is evidence of violations of the human rights of children and women, and some cases of harmful and degrading practices have been documented [3].

Against this background, an interdisciplinary group of ethicists, researchers and practitioners convened in Israel to discuss the need for international governance of IMAR (for a list of the participants, see Additional file 1). Israel is a country in which MAR is practiced extensively with almost unlimited public funding, resulting in the highest per capita rates of usage worldwide [4]. Courts recognize a constitutional right to parenthood, and the Knesset, Israel’s parliament, has enacted legislation that establishes a regulatory system of bureaucratic approvals for various third-party MAR practices, based on statutory criteria of eligibility. Israel’s Surrogate Mother Agreements Law (1996), was the first in the world to allow commercial surrogacy under the supervision of a statutory committee [5, 6]. The Egg Cell Donations Law, 2010 enacted a similar system [7]. Nonetheless, despite liberal domestic law, IMAR practices have grown rapidly in recent years, mainly because of restrictions on access to domestic surrogacy for same sex couples [8] and a shortage of healthy women who are willing to provide their eggs for the treatment of couples and singles in need [9]. Although the Egg Cell Donations Law allowed ‘donations’ from healthy volunteers and compensation for their effort, it did not alleviate the ‘shortage’ of egg cells in the country. Therefore Israel, despite its relative small population size, has become an important site for gathering information regarding the complex mechanisms of IMAR usage, and indicates the urgent need for agreements and regulations that will ensure the health and well-being of all collaborators.

This document is based on our collective experience and knowledge. Our discussions revealed differences of opinion that reflect multiple perspectives on the complex issues of IMAR, even among professional researchers who are all committed to a human rights based approach. We discovered, *inter alia*, different concepts of autonomy, different views as to the degree to which the State should interfere in agreements between consenting adults, and different opinions as to the proper balancing of competing rights and values. But by all indications the issues are here to stay, and will likely grow as new business opportunities emerge to bring to the IMAR market controversial technological innovations, such as the recent developments of mitochondrial replacement therapy, and whole genome sequencing or CRISPR-Cas9 (‘gene editing’) for embryos [10].

The purpose of this paper is to call for a discussion of the need for IMAR international governance at multiple levels – the international community, nation states, professional organizations and civil society – as market forces lead the proliferation of reproductive technologies for individuals of means.

The subject matter is extremely controversial. Questions of children’s legal parentage and nationality in transnational surrogacy have been on the agenda of the Hague Conference on Private International Law for several years. A comprehensive document prepared by its Permanent Bureau in 2014 notes the diversity in states’ domestic law regarding the establishment of legal parenthood, and emphasises the importance of focusing on building bridges between legal systems based on internationally established common principles, rather than the harmonisation of substantive laws concerning legal parentage [11]. Yet discussions there have yet to resolve the divergent views on the legal status of children born in cross-border situations that circumvent legal prohibitions in the parents’ country of origin [12].

What is more, public international law aspects of IMAR practices that are similar to the field of organ transplant tourism, such as trafficking in human beings and body parts [13] – are not within the mandate of the Hague Conference, and have not been addressed so far by any other relevant international forum.

The goal of this paper is to set an agenda for discussion, to identify areas of concern, to suggest good practices that might alleviate some of the most grievous consequences of an unregulated IMAR market, and to describe points of disagreement that require further exploration. The paper concludes with a call for action at the international, national and professional levels within the framework of a feminist ethic of care for all involved individuals, including the children and the women who assist in bringing them into the world. We believe that continuing discussion and deliberation will eventually lead to clarity as to the promotion of fair practices, the prevention of human rights violations and the criminalization of extreme abuses.

## Terminology

Much of the literature on the subject of IMAR refers to “cross-border reproductive care”. This reflects the viewpoint of individuals suffering from infertility who need and seek access to medical treatment which is either unavailable or unaffordable in their countries of origin. We chose to use the term “inter-country MAR” because it accommodates the viewpoints of all involved individuals, including the third-party reproductive collaborators. Moreover, we refrain from using the term ‘care’ which carries underlying assumptions of altruism and empathy, which is not necessarily present in the medical interventions involved in medically assisted reproduction, especially when involving third-party reproductive laborers.

Similarly, much of the literature addressing domestic issues refers to ART (“assisted reproductive technology”), rather than MAR (“medically assisted reproduction”). We chose the latter, because it reflects the human activity of reproduction, whereas the former focuses on the technology.

Human reproduction by its very nature involves the collaboration of human beings, in particular women, whether or not medically assisted, and whether or not it involves third-party individuals. The notion of collaboration implies respect for all those assisting in the birth of the child. Because reproduction is essentially collaborative, we use the term “third-party collaborators” to denote the genetic progenitors (gamete “providers”) and the women who carry pregnancies and give birth to children (“surrogate mothers“) for other individuals whom we call the “intended parents”.

The term “providers” is used for those whose gametes (egg cells and sperm) are used in the reproductive collaboration, so as to preserve the term “donors” for those who act altruistically in non-commercial relationships, and “procurement” rather than “donation” is used for the same reason.

## Ethics and human rights

Our theoretical approach is a human rights based ethics of care and responsibility. As opposed to transnational transplant medicine, where professional self-governance provided the basis for an emerging consensus in international law, IMAR remains an unregulated market driven by the desire of prospective parents for a family and a healthy child and the profit making interests of medical entrepreneurs and the biotechnology industry [14]. At the moment, there are no internationally accepted ethical principles or clinical standards for the quality and safety of MAR interventions. The distribution of scarce human bio-resources is done according to ability to pay rather than considerations of justice or solidarity [14, 15]. There are no mechanisms in international law for transparency and accountability, nor for regulatory oversight in case of human rights violations. And lastly, there is no understanding of what differentiates legitimate cross-border medical travel from reproductive trafficking, and no criminal justice redress for instances of exploitation, deception and coercion [13].

MAR has brought many blessings to numerous individuals worldwide, but in some cases this has incurred harm to other individuals. The main approach of this paper is to suggest good practices so as to avoid harm to children and third-party women and men. But we also acknowledge known cases of such harm and argue for the need to prohibit the most grievous harmful practices as tantamount to reproductive trafficking.

Our view comes from a commitment to an ethic of care and responsibility, respect and solidarity towards all the adults involved in IMAR collaborations, concern for the rights and well-being of the resultant children, and a commitment to inter-generational justice and responsibility for the heritage of humanity that we pass on to future generations [16]. We align our call with concerns brought to the fore by feminist scholars in recent decades [17, 18], while also recognizing the agency of reproductive labourers and the need for their involvement in the discussion, as suggested by ethnographies of the reproductive trade [19–21]. Our view is that the activity of reproduction is intrinsically dependent on collaboration with others, and the relational context of this activity should be acknowledged so as to avoid the objectification of third-party collaborators. We believe that it is in the child’s best interests to be born from and into relationships, however short- or long-lived, that are based on respect, reciprocity, trust and integrity between intended parents and third-party collaborators [22].

The working group reaffirmed its commitment to values of fundamental human rights and the dignity and worth of the human person. These include the equal rights of men and women, regardless of race, class, marital status and sexual identity. Multiple instruments of international human rights law contain principles and rules that are relevant to IMAR, including the right of adult men and women to found a family, the right of women to reproductive health, the right of persons to autonomy in medical decision making, and the right of children to identity, parentage and nationality [23–26]. (For further detail on relevant instruments of international human rights law, see Additional file 1).

However, none of those instruments address the potential for the exploitation, coercion and deception of women as providers of reproductive services and resources. There is a consensus that human beings and their body parts cannot be the subject of commercial transaction and financial gain [27–33]. But issues of third-party IMAR practices are not addressed in the relevant instruments that prohibit servitude and trafficking in human beings and organs, while instruments on tissues and cells typically exclude the cross-border transportation of human sperm, egg-cells and embryos [13].

A common argument in defence of the MAR market derives from the principle of personal liberty and freedom of contract [34]. However, much as personal liberty is inalienable and cannot extend to the right of an individual to sell one self into slavery [35, 36], and much as freedom of contract is constrained by considerations of morality and public policy, the freedoms and rights of infertile persons to establish a family through IMAR may be subject to limitations for the purpose of meeting just requirements of morality and public order in the global marketplace. Such restrictions are necessary and justified out of respect for the rights and freedoms of both the children and the third-party women who provide their bodily services and resources to assist in bringing them into the world [37].

## Areas of concern

IMAR is a particular form of medical tourism but it raises concerns beyond those which are typical to critiques of general medical tourism practices, such as quality of care, and the issues of affordability and accessibility which concern distributive justice in two-tier health systems [38–40]. Unlike most situations of cross-border medical care, IMAR also involves the use of another (non-patient) person’s body as a means of medical “treatment”. In this it is similar to transnational organ transplant procedures. We therefore believe that our discussion aligns better with bioethical discussions of organ transplant medicine in cross-border settings. Furthermore, IMAR also involves the creation of a child, and thus aligns with inter-country adoption as well as the literature examining the commodification of human bodies and intimacies more broadly.

### Intended parents

Despite the many benefits of MAR in alleviating infertility, the proliferation of this technology has led to multiple new forms of associated suffering. Despite the many children born to otherwise infertile persons by means of MAR, infertility as such continues to be experienced as distressful and socially stigmatized. Childlessness may be remedied, but people want more than one child. In addition, infertility treatment itself is physically and financially taxing and often entails multiple unsuccessful cycles. Emotional harms associated with infertility treatment include anxiety and grief, as well as stress and disruption of spousal relations, shame and blame, anger and depression, low self-esteem and stigma [22]. The suffering of the thwarted desire for a child may be aggravated by limitations on access to treatment for couples and individuals in need due to the lack of available or affordable services. In 2011 only 48 out of 191 member States of the World Health Organization had IVF facilities. Among those that do, many do not have insurance schemes for reimbursement for MAR treatment [41].

At the same time, success rates remain relatively low: pregnancy rates per treatment cycle are around 35%, with around 25% chance of a live birth per treatment cycle [42]. Risks to the health and well-being of women from preparatory hormonal treatment, egg retrieval and multiple embryo-pregnancies are well known [43]. Multiple-embryo pregnancies are also associated with premature delivery and low birth weight new-borns. Moreover, infertility patients seeking treatment outside their home countries might be at increased risk due to lack of control over quality and safety standards; the absence of counselling; inadequate information about possible health risks; and increased exposure to incompetence, negligence and recklessness [44].

In the case of third-party IMAR, intended parents are vulnerable to disinformation and exploitation by intermediaries in foreign countries. Added risks include uncertainty as to the source of gametes or embryos, and financial extortion by intermediaries who might also obstruct attempts to contact, deal directly and form a relationship with surrogate mothers. In addition there are numerous bureaucratic hurdles to establishing parentage and returning home with the children [45].

### Third-party collaborators

As for egg cell providers and surrogate mothers, a major concern is the exacerbated risk of harm from medical interventions because of a double standard of care, that is, care that is centred towards the paying customer rather than the surrogate’s or egg donors’ medical needs, as well as emotional and financial harm due to unequal relations of power between third-party collaborators and commissioning parents, and the potential bias of mediators and professionals within the IMAR industry.

Physical risks to egg cell providers include the pain and discomfort of daily hormonal injections and harmful side effects, including anaesthesia complications, ovarian hyper-stimulation syndrome, damage to reproductive organs and post-retrieval complications of surgery [46]. Recruitment advertisements on university campuses do not mention these risks, and the women might undergo excessive repeat procurement cycles without being informed of the risks involved [47]. Although there has been no systematic medical tracking of the effects of egg cell procurement on otherwise healthy young women, there is no evidence base for the safety of the procedure in the medical literature, and there are controversies regarding long-term risks of breast and ovarian cancer [48]. Anecdotes abound of loss of fertility, stroke, cancer and premature death, while psychological risks of detachment from resultant children might arise years later [49, 50].

The potential for exploitation and deception of women who provide egg cells for others is illustrated by a case from Israel that came to light in 2000: a leading fertility expert confessed in professional disciplinary proceedings to having submitted patients to excessive hormonal stimulation, retrieving dozens of eggs from single treatment cycles, and using these eggs in the treatment of large numbers of recipients, without the knowledge of the providers. In one case he retrieved 256 eggs from one woman and used 181 of them to treat 34 others [7, 51].

Research from Israel on domestic surrogacy agreements reveals similar vulnerabilities of third party women to a double standard of medical care and disinformation, and also to emotional harms and violations of privacy and autonomy. Israel provides a rich data source on commercial surrogacy, since the Surrogate Mothers Agreements Law (1996) requires approval of any surrogacy contract signed and performed within the country. It is therefore possible to know exactly how many surrogacy contracts were signed since 1996 and their outcomes. Data collected from official records of approved surrogacy agreements show a relatively high rate of multiple births. Notably, less than 40% of the agreements actually result in the birth of children, and commercial practices often do not pay women for unsuccessful treatment cycles, failed attempts to become pregnant or spontaneous miscarriage of a pregnancy, while the women report a heavy emotional toll of failure [8, 52]. Nor are the women remunerated fairly for the time and energy they invest in the process of applying for bureaucratic approval, including intrusive mental and physical diagnostic procedures [52]. In the case of a successful pregnancy, agreements typically restrict the surrogate’s lifestyle and personal freedom, with obligations to refrain from sexual intercourse, not to smoke, not to eat certain foods, and a requirement to obtain permission from the intended parents to travel outside the country, thus limiting their personal autonomy beyond what would be expected in the case of women carrying their own child. Surrogate mothers, like egg providers, appear to be motivated by both financial interests and noble altruistic sentiments, and report forming an emotional attachment with the intended parents during the pregnancy, often with a sense of self-worth as a result of this relationship, which allows them to experience the process as an act of heroism rather than exploitation [53]. But once they deliver the child this relationship might be severed abruptly and surrogates report having little control over the process of separation after having given birth [8].

The vulnerability of third-party reproductive collaborators to harm is exacerbated in inter-country settings due to structural inequalities, geographical distance and cultural gaps. There is limited quantitative data, because IMAR takes place in a private market. But social science studies, human rights reports and documentary films – mostly about India – indicate patterns of exploitation, deception and coercion that might amount to human trafficking [13]. Cases in which women have been recruited to travel and tricked or forced into working as surrogates have been documented in Guatemala, Poland, Myanmar and Thailand [54]. In more routine cases, intended parents may set in course a process. marketed and facilitated by intermediaries, that culminates in the birth of a child without having met or seen their third-party collaborators. The relative invisibility of resource providers to those who purchase gametes or surrogacy services in these markets, due to language and cultural barriers as well as geographical and social distancing, is a factor that objectifies them and diminishes concern for their well-being [22, 45].

Egg providers are typically recruited to be a racial match with intended parents, but do not receive any information about their identity. International surrogacy agencies working from Israel recruit women from countries such as the Ukraine and South Africa, offering them a “reproductive tourism” package that includes egg “donation” and a holiday in India, Thailand or Nepal. Women in India will provide eggs for intended parents who are Indian, whether residing in or outside the country. These women might also work as surrogates and as human subjects in clinical trials. One woman who provided eggs recounted that the hospital told her to get lost after the retrieval procedure and refused to give her any medical record of the intervention [55].

Surrogacy practices in India incur impaired autonomy in decision making about the pregnancy: choices about the numbers of embryos implanted, termination of pregnancy, lifestyle during pregnancy, and interventions during labour and delivery such as c-section will be made by the intended parents and medical professionals. The literature describes deprivations of liberty (confinement in hostels for the duration of the pregnancy, with controlled nutrition and limited family visits), violations of patient autonomy and bodily integrity (non-consensual abortions, routine c-sections) and exploitation of maternal labor (multiple embryo implantations, and breast milk nursing pending the late arrival of intended parents). Social harms include stigmatization [3, 56–59].

In many cases, surrogate women are required to leave their homes and live in dormitories or housing providing by the surrogacy clinics and agencies. These practices have been documented in India [55, 59–61], Nepal (Eyal H, Samama E, Shalev C. Transnational surrogacy and the earthquake in Nepal: a case study from Israel. In: Miranda Davis [ed], Global Babies: Transnational Surrogacy and the New Politics of Reproduction (Zed Books, forthcoming 2017)) and Russia [62]. In such dormitories or housing arrangements, surrogates are fed and monitored around the clock by the clinic personnel, and in extreme cases are not allowed to exit the site or engage in physical activity [3, 55, 59, 60]. One of the narratives is about a surrogate awaiting the arrival of the intended parents, an Indian couple from Canada, after giving birth to twins. After delivery, she expresses breast milk to feed the babies. Ten days after the birth the parents have still not come and she ventures into the infant unit to see the babies. As time goes by and the parents still do not come, she starts physically taking care of the infants and names them. The couple arrive only three weeks after the babies were born [60].

While the standard of care for MAR in developed countries now discourages the implantation of multiple embryos because of the risks to the health of the pregnant woman and to premature newborns, it is often practiced in IMAR [61]. Surrogates are usually offered a bonus payment for carrying and giving birth to twins, but if more than two embryos develop they are expected to undergo a procedure of embryo reduction to abort the excess one [3, 61, 63].

Accounts of intended parents from Israel stranded in Katmandu with their newborns at the time of the earthquake there in May 2015, indicated relatively large numbers of twins and premature births. The clinic there had a 100% rate of c-sections, which the women were told was the “best way” to give birth – yet another instance of a double standard of medical care. Of course, c-section allows for control over the time of the birth of the child, so that intended parents can plan travel accordingly. According to the accounts of intended parents, their expectation was that the children would be born at 36 weeks, rather than 40, which is the norm (Eyal H, Samama E, Shalev C. Transnational surrogacy and the earthquake in Nepal: a case study from Israel. In: Miranda Davis [ed], Global Babies: Transnational Surrogacy and the New Politics of Reproduction (Zed Books, forthcoming 2017)).

A business model that guarantees an end product and caters to the preferences of customers has also led to what is known as ‘twin’ or ‘twibling’ surrogacy, where two surrogate mothers are hired at the same time in order to maximize the chance of a live birth [64]. At a ‘surrogacy fair’ in Israel, in February 2013, attended by 15 surrogacy agencies from Israel and the USA, one agency offered potential customers a track of ‘parallel pregnancies’ in which several women would carry pregnancies for a single prospective family, so as to increase the chance of producing a child within a certain time frame. It was implied that if the achieved pregnancies exceeded the planned number of children, the ‘excess’ pregnancies would be terminated. The women carrying the aborted pregnancies have no say in the decision. They might be deceived and told that there is a medical indication related to the health of the foetus. According to one surrogacy agent operating in Eastern Europe, under their contract surrogates might not be entitled to payment for their services if a live child is not produced.

### Children

While the number of children conceived as a result of inter-country surrogacy and other IMAR arrangements has increased dramatically in recent years, there have been certain extreme cases of child trafficking in which the babies have become commodified as a marketable product of exchange [54]. For example, the surrogacy industry in India has also produced ‘extra’ babies, either because excess pregnancies are carried to full term or because intended parents do not claim the children they ordered. At this point the abuse of surrogate mothers turns into baby selling. In a recent documentary, one journalist went undercover to meet a surrogacy agent who claimed there were ‘extra’ babies being sold on the black market, and there and then offered to sell her one on the spot [65, 66].

In February 2012, Theresa Erickson, a USA attorney specializing in reproductive law was sent to prison for her role in an international baby selling scheme. In her guilty plea, Erickson admitted that she and her conspirators used surrogate mothers to create an inventory of unborn babies that they would sell for over $100,000 each. They accomplished this by paying women from the USA to travel to the Ukraine, to become implanted with ‘donated’ sperm and eggs. If the women sustained their pregnancies into the second trimester, the conspirators offered the babies to prospective parents by falsely representing that the unborn babies were the result of legitimate surrogacy arrangements, but that the original intended parents had backed out [67, 68].

A recent decision of Israel’s Supreme Court ruled that a genetic connection between the child and at least one of the intending parents is needed in order to rule out child trafficking. The case concerned a single woman who arranged for the fertilization of embryos with the sperm of an acquaintance and the egg cell of an anonymous provider from South Africa. The woman’s niece carried the pregnancy for her after undergoing embryo implantation in India, and gave birth to the child in Israel. The woman then petitioned the court for a parenting order, which she was denied. The court reasoned that the law does not recognize parentage that is purely contractual, and making babies cannot be left to simple agreement for the creation of a product [69].

In other cases children born of IMAR have been rendered parentless and stateless, in violation of the rights of the child to nationality and parentage under article 7 of the Convention on the Rights of the Child [70, 71]. The baby is born in one country on the basis of an agreement with the intended parents who live in another, and they need travel documents to bring the baby home. But conflicts of domestic law can arise between the two jurisdictions as regards the determination of legal parenthood. In one case, intended parents from the UK had a child from surrogacy in the Ukraine. Under the law in the UK the surrogate and her husband would be considered the legal parents, while under the law in the Ukraine the child’s legal parents were the intended parents, so they could not adopt the child to be recognized as her parents under UK law.

In another case, the European Court of Human Rights found that France had violated the right of children born of international surrogacy to respect for private family life under Article 8 of the European Convention on Human Rights, by denying the parent–child relationship that had been legally established in the USA, where the children were born. The decision concerned two couples from France who had children biologically related to the male partner by means of a surrogacy agreement in the USA, where the legal parent–child relationship had been recognized. The French authorities refused to enter the birth certificates in the French register of births, because that might be seen as giving effect to a surrogacy agreement that was null and void under French law on grounds of public policy [72].

Other cases have involved abandonment of the children. For example, an infant was born in India in 2010 to a married couple from Japan, who had divorced during the course of the pregnancy. Neither the Indian birth mother nor the Japanese intended mother wanted the child. At the time Japanese law did not recognize surrogacy and the intended father could not adopt the child under Indian law because he was now single. The baby’s paternal grandmother took responsibility for the baby but they were stranded in India for six months while trying to overcome the legal hurdles to obtaining travel documents (Margalit, Yehezkel. From Baby M to Baby M(anji): Regulating international surrogacy agreements. J Law Policy. Forthcoming) [71, 73]. A more recent and much publicized case was that of Baby Gammy born as a twin in Thailand in 2014 to an Australian intended couple. Gammy had Down’s syndrome and a congenital heart condition, and the intended parents took his healthy twin sister home while abandoning him. The Thai surrogate mother took responsibility for Gammy, and succeeded eventually in obtaining Australian citizenship for the child and rights of access to health care in Australia [74].

Yet another crucial issue concerns the right of the child to identity, or the right to know the circumstances of one’s birth and origin. This has both psychological and health-related aspects. Medical documentation about genetic progenitors is obviously relevant to informed health care decision-making, but the right to know has more far-reaching meaning as is evident from the growing support for the moral right of donor-conceived children to know their genetic origins [75]. It is a key facet of the child’s sense of self-identity and his or her connectedness with heritage and kin, be they the genetic father and mother, the woman who gave birth, or part-siblings. But in IMAR no one has the legal obligation or responsibility to keep records of gamete providers and surrogate mothers. This erases the identity of the third-party collaborators while compromising the child’s ability to learn of his or her circumstances of birth later in life.

## Discussion

Arrangements between intended parents and third-party reproductive collaborators create a special kind of agreement that needs regulation so as to protect the interests of all the involved persons: the intended parents, the third-party collaborators and the children. In inter-country settings, under conditions of geographical distance and cultural disparity, the for-profit motivation of medical entrepreneurs and intermediary agents exacerbates the potential commodification of women and children. The unregulated market of IMAR involves the commercialization of human reproduction and transforms the personal and intimate nature of reproductive relations into contractual and labour relations. Considering also foreseeable technological developments that would allow the genetic selection and modification of human embryos, there are profound concerns about the moral limits of markets and the impact of market-driven repro-genetic technology on the future of humanity and the very nature of the human species.

In inter-country settings, the current lack of professional self-governance and the absence of internationally accepted clinical-ethical guidelines for MAR are conducive to potential abuse of third-party women who collaborate to fulfil the desire of others to have a child - throughout the process of egg cell extraction, fertilization, impregnation, implantation, gestation, miscarriage, labour, delivery and post-birth nursing and care. These women are often treated according to double standards of care for invasive medical interventions, ethical standards of consent to treatment are not observed, and decisions about the medical interventions they undergo are often made by others. At times they have no direct contact with intended parents and do not even know who they are. Intermediaries perform a necessary social function in mediating between individuals seeking MAR services outside their countries of residence. However, the commercial nature and profit-seeking motivation of this function create conditions that are conducive to exploitation.

There is evidence that unregulated IMAR can lead to grave violations of women’s dignity and human rights, as described above. In extreme circumstances, abuses might even amount to human trafficking, in the sense of the appropriation and control of women and children as commodities. Thus, there is an urgent need to conceive a governance regime for the unregulated IMAR market so as to ensure safe and fair practices, minimize harms and prohibit abuses.

IMAR need not necessarily be abusive or incur violations of human rights. Lessons learned from countries in which MAR is regulated indicate elements of a good practice model by which new forms of multi-parent families can be established on the basis of mutual respect, intimacy and relationship between intended parents and reproductive collaborators, with support and counselling for all the involved adults throughout the process. Most of the participants in the working group of Israeli experts thought that open relationships between third-party collaborators and the children and their families could be encouraged, and the altruistic motivations of third-party collaborators could be acknowledged even if they are also paid for their work. Some thought that fully altruistic arrangements should be seen as best practice, i.e., where the egg donor or surrogate mother is a relative or friend of the intended parents. In such case, however, it would be necessary to ensure that the women are not induced to collaborate as a result of family or social pressure, and that they are fully informed of the risks involved in the process and provided with compensation if these risks should materialize.

One view in the literature [45] is that ideally countries might aim to adopt a policy of national self-sufficiency so as to meet domestic needs for MAR, including third-party reproductive collaborations, and to minimize disincentives to local providers of gametes and surrogacy services such as lost wages, costs of travel and out-of-pocket expenditures. Nonetheless, international governance is needed since it is improbable to assume that the global market will disappear.

First and foremost, international bodies and nation states should recognize new forms of family and should guarantee the child’s right to parentage, nationality and identity. Some of the working group participants considered that responsibility for the welfare and best interests of children born of IMAR should be paramount. Therefore, in case of conflicts of law as regards the child’s parentage, the default presumption should be that the country of birth is *parens patriae*, in accordance with the principle of subsidiarity. Likewise, this view suggested that children born of IMAR should have a right to nationality in both the country in which the intended parents are nationals and the country of birth. This would prevent the child from being rendered stateless in case of dispute about the child’s parentage and make it the responsibility of both countries to care for children born of reproductive collaborations initiated by their nationals, or within their jurisdictions.

As for the right to identity of children born of IMAR, i.e., the right to know the origins of conception and circumstances of birth for both medical and psychological needs, the consensus among the working group was that the medical professionals who administer the procedures that result in the child’s birth should have a legal obligation to preserve identifying information about the third-party collaborators.

However, there was disagreement about whether or not children have a right to know the identity of their genetic progenitors, as in adoption, and whether or not they have a right to know the identity of their gestational mother. One view was that the child has a medical interest in knowing the identity of the genetic mother, but does not have any interest in knowing the identity of the woman who carried the pregnancy and gave birth if there is no genetic relation between the two. Others considered this view – that genetic motherhood is of greater value than gestational motherhood – to be an expression of genetic essentialism and materialism, and to reflect a gender bias since genetic parenthood is the only form of biological parenthood for the male of the human species, as opposed to the female form of biological parenthood which can be either genetic or gestational. According to this point of view, epigenetics show that the gestational environment has significance for the child’s development, and female parenthood emphasizes the nurturing aspect of human relationship.

What is more, the third-party collaborators also have an interest in whether or not their identifying information is preserved and made accessible to the children [76]. The issue of the anonymity of third-party collaborators is controversial. Its origin is in the practice of sperm ‘donation’. Recognition of the children’s interest in knowing the identity of their fathers has led some jurisdictions to legislate a right to disclosure for ‘donor’ offspring similar to the law of adoption. The members of the working group were divided as to whether a similar scheme should apply to egg cell procurement in inter-country settings. Some considered that anonymity was a compromise of parental responsibility and should be discouraged. Others considered that it would not be beneficial if disclosure of identifying information led to a decrease in egg cell provision, and that potential providers should be given the choice as to whether to be anonymous or identifiable when the child reached the age of majority.

In any event, most participants thought there was a difference between egg cell procurement and surrogacy, and there was widespread agreement that anonymous surrogacy should not be allowed because it violates human dignity. From the point of view of the woman who carries the pregnancy and births the child, anonymity and the erasure of any identifying information renders her invisible, and is a means of objectification, commodification and instrumentalization that dehumanises the person as a mere vessel. It is therefore important to make sure the gestational mothers are present as human beings, and they have a right to be acknowledged as having birthed the children and to choose whether and how to have ongoing contact with them.

There was also substantial agreement about drawing red lines of extremely harmful IMAR practices that should be prohibited as criminal offences under both domestic and international law. Drawing parallels from international documents on organ transplant trafficking [27, 77, 78] these offences might include:

- medical interventions in third-party collaborators without the free, informed and specific consent of the patient;
- the use, storage and transportation of illicitly procured human reproductive cells and embryos;
- the commercial brokerage of IMAR services, including solicitation, advertisement and recruitment of sperm and egg donors and surrogate mothers for financial gain (i.e., advertisement and brokerage involving payment);
- the implantation of human embryos outside of the framework of the domestic regulatory system;
- the solicitation of gamete donors and surrogates to cross national borders, for the purpose of evading local protective regulations or undermining the rights of reproductive labourers in their country of origin;
- the offer or receipt by health care professionals of any undue advantage in connection with illicit IMAR practices.

In general, countries of origin and destination should take responsibility to quell the cross-border abuses of women and children perpetrated by nationals in circumvention of domestic law. Ideally, they should not allow a double standard of intra- and extra-territorial legality, and would exercise extra-territorial jurisdiction over offences committed by or against nationals or other individuals who are resident within their jurisdiction, in contravention of domestic restrictions on access to MAR [79, 80]. In this respect, the question whether intended parents should be penalized for circumventing domestic laws needs further consideration because it involves possible stigmatisation of children with “new illegitimacy”. However, intermediary agencies should be held responsible.

Many participants in the working group took the position that commercial intermediary agencies should be banned and replaced by non-profit organizations with the capacity to provide professional counselling, similar to the model of the Hague Convention on Inter-Country Adoption, 1993. The group was divided as to whether individuals representing IMAR agencies currently operating out of Israel should be invited to participate in the process of deliberation about the need for international governance. Some thought that their experience and knowledge of the field would be a valuable contribution to the discussion, and that they too should adopt a code of business ethics, while others considered that commercial interests would skew the debate.

## Call for action

In light of all the above, it appears to be time for a system of international governance that addresses the challenges that IMAR presents. The system should be based on human rights and promote universal access to MAR for the treatment of infertility through the sharing of knowledge, transfer of technology and publicly funded services [81], and be based on a combination of three existing models of regulation: (1) an international mechanism for monitoring IMAR practices; (2) inter-country adoption; and (3) trafficking in human beings, organs and tissues.

Existing mechanisms of international monitoring, such as those operating within the UN human rights treaty bodies, or for public health purposes within the WHO Framework Convention on Tobacco Control, 2003 might be adapted to the context of IMAR so as to guarantee the collection and reporting of transparent data as follows:

- To report on adverse events affecting the health and well-being of third-party women and children born of IMAR;
- To ensure the provision of post-procurement, post-implantation and post-birth clinical follow-up care for third-party women;
- To gather epidemiological data on IMAR and enable the conduct of longitudinal studies on the health and well-being of children and of third-party women;
- To collect information for the traceability of human reproductive cells and embryos at both national and international levels, so as to guarantee quality and safety in the interests of public health [27, 30, 31, 33].

Regulatory measures drawn from the model of the Hague Convention on Inter-Country Adoption would require the accreditation of not-for-profit IMAR agencies, so that services involving women as third-party reproductive collaborators are provided equally and fairly with due transparency and accountability. Such measures might also establish designated central authorities for maintaining a national registry of IMAR children, gamete providers and surrogates, in order to guarantee the right of the children to access information regarding their genetic origins and circumstances of birth.

A regulatory model based on international norms concerning trafficking in human beings, organs and tissues would likewise establish a transparent system of national oversight by means of competent not-for-profit national authorities with overall responsibility and accountability for IMAR practices involving nationals, including traceability [33]. It would also ensure standards of provider and recipient safety through the accreditation of MAR centres for gamete procurement and embryo implantation, and establish rules of distributive justice that govern the transparent allocation of and equitable access to limited medical services and human resources, including human reproductive cells, according to evidence-based clinical guidelines. An anti-trafficking approach would call for cooperation between countries of origin, transit and destination to adopt necessary measures to prevent, protect and prosecute the exploitation, deception and coercion of third-party reproductive collaborators and the sale of children.

We, therefore, call upon the United Nations and other inter-governmental organizations and their agencies, international human rights bodies and international professional associations, nation states and civil society, and upon all concerned individuals – jointly and severally, to take all possible measures to respect, protect and fulfil the human rights of women and children involved in IMAR, including the following:

- To take appropriate measures, at both national and international levels, to prevent practices which lead to the commodification of children and women;
- To criminalise IMAR practices which involve the sale of human beings and their body parts and resources, including human reproductive cells and embryos;
- To prohibit IMAR practices that involve the exploitation, deception and coercion of third-party women and men, and other violations of equity, justice and respect for their human dignity and human rights [32], regardless of the victim’s consent [79, 82];
- To provide medical, psychological and social care for the short- and long-term effects of MAR on the physical and emotional health and well-being of third-party women who provide their reproductive resources for the benefit of others, and for the recovery of victims of exploitation, deception and coercion, and reproductive trafficking [83].

Perhaps most importantly, the working group considered that medical professionals are key links in the IMAR industry, without whose involvement none of the harmful practices would be at all possible. As opposed to the field of organ transplantation, in the area of reproduction professional organizations have not laid down clinical standards of efficacy, quality and safety, and have not taken a leadership role in terms of ethical self-governance.

We therefore call upon professional medical associations to take a leading role of self-governance in advancing the international regulation of IMAR, and to establish clinical and ethical guidelines that set universal standards of respect and care for women undergoing MAR treatment worldwide. The medical profession should also take responsibility to ensure the traceability of human gamete donations and embryo implantations, and to preserve information necessary to realize the right of the child to know his or her origins. And last but not least - to adopt standards of conduct that sanction health care professionals who are involved in illicit IMAR practices.

## Additional file

Additional file 1: A. Participants in the ERIMAR working group. B. Relevant articles of international human rights legal instruments. (DOCX 19 kb)

## Abbreviations

IMAR: Inter-country medically assisted reproduction
MAR: Medically assisted reproduction

## Footnotes

1 Since in most cases eggs and sperm are provided for a cost, and providers are financially compensated for their genetic materials, we refrain in this article from the common usage of the term “donation” in reference to these practices, and prefer the more neutral and accurate term “provision” or “providers”.
