# Supplementary material for: Ethics and regulation of inter-country medically assisted reproduction: a call for action
Source: Isr J Health Policy Res. 2016 Dec 7;5:59. doi: 10.1186/s13584-016-0117-0 (PMC5142386; doi:10.1186/s13584-016-0117-0)
Supplement: Additional file 1: — A. Participants in the ERIMAR working group. B. Relevant articles of international human rights legal instruments. (DOCX 19 kb) [file 13584_2016_117_MOESM1_ESM.docx]

## Appendix A: PARTICIPANTS IN THE ERIMAR WORKING GROUP

**Last name First name Affiliation Expertise**

Bassan Sharon Tel Aviv University Reproductive health law

Eldar-Geva Talia Hebrew University Obstetrics & gynecology

Shaare-Zedek Medical Center

Eyal Hedva Hebrew University Public policy

Golan Orit Bar Ilan University Philosophy

Hashiloni-Dolev Yael Tel Aviv-Yaffo Academic College Sociology

Landau Ruth Hebrew University Social work

Laufer-Ukeles Pamela Bar Ilan University Family law

Leibel Mihal Atzum Task Force on Human Trafficking

Lepicard Etienne Ashkelon Academic College History of medicine

Karni Tamar Israel Medical Association Medical ethics

Margalit Yehezkel Ono Academic College Health law

Moreno Adi University of Manchester Social anthropology

Samama Etti Ben Gurion University Medical technology

Schiff Anne Independent scholar Reproductive law

Schuz Rhona Sha’arei Mishpat Law School Private international law

Segal Peretz Ministry of Justice, Israel Inter-country adoption

Shalev Carmel Haifa University Bioethics & law

Shmueli Merav Ministry of Justice, Israel Human trafficking

Triger Zvi College of Management Law & gender

Zafran Ruth Inter-Disciplinary Center Family law

## Appendix B: RELEVANT ARTICLES OF INTERNATIONAL HUMAN RIGHTS LEGAL INSTRUMENTS

Rights of adults

- Men and women of full age have the right to found a family;^[[1]](#footnote-1)^
- The autonomy of persons to make decisions, while taking responsibility for those decisions and respecting the autonomy of others, is to be respected;^[[2]](#footnote-2)^
- Any medical intervention may be carried out only with the prior, free and informed consent of the person concerned, based on adequate information;^[[3]](#footnote-3)^
- In applying medical technologies, individuals of special vulnerability should be protected and the personal integrity of such individuals respected;^[[4]](#footnote-4)^
- Everyone has the right to just and favourable conditions of work;^[[5]](#footnote-5)^
- The right of women to protection of health and to safety in working conditions, including the safeguarding of the function of reproduction;^[[6]](#footnote-6)^
- The rights of women to appropriate services in connection with pregnancy, confinement and postnatal care;^[[7]](#footnote-7)^

Rights of children

- All children, whether born in or out of wedlock, have the right to enjoy the same social protection;^[[8]](#footnote-8)^
- The child is protected against all forms of discrimination on the basis of the status or activities of the child’s parents;^[[9]](#footnote-9)^
- The child shall be registered immediately after birth and shall have the right to acquire a nationality and as far as possible, the right to know his or her parents;^[[10]](#footnote-10)^
- The child has the right to preserve his or her identity.^^[[11]](#footnote-11)^^

Trafficking in human beings and body parts

- Human beings and their body parts cannot be the subject of commercial transaction and financial gain;^^[[12]](#footnote-12)^^
- No one shall be held in slavery or servitude;^[[13]](#footnote-13)^
- All forms of slavery, servitude, bondage and trafficking in human beings are prohibited,^^[[14]](#footnote-14)^^  including trafficking in women^[[15]](#footnote-15)^ and children and the sale of babies.^[[16]](#footnote-16)^

1. Universal Declaration of Human Rights, 1948 – Article 16. This article did not foresee the multi-parent extra-marital families that have resulted from MAR, but the principle of the free and full consent of the intended spouses applies *mutatis mutandi* to prospective parents and reproductive collaborators. [↑](#footnote-ref-1)
2. UNESCO Universal Declaration on Bioethics and Human Rights, 2005 – Article 5. [↑](#footnote-ref-2)
3. UNESCO Universal Declaration on Bioethics and Human Rights, 2005 – Article 6. [↑](#footnote-ref-3)
4. UNESCO Universal Declaration on Bioethics and Human Rights, 2005 – Article 8. [↑](#footnote-ref-4)
5. Universal Declaration of Human Rights, 1948 – Article 23(1). [↑](#footnote-ref-5)
6. The Convention on the Elimination of all forms of Discrimination Against Women, 1979 – Article 11(1)(f). [↑](#footnote-ref-6)
7. The Convention on the Elimination of all forms of Discrimination Against Women, 1979 – Article 12(1). [↑](#footnote-ref-7)
8. Universal Declaration of Human Rights, 1948 – Article 25. [↑](#footnote-ref-8)
9. The Convention on the Rights of the Child, 1989 – Article 2(2). [↑](#footnote-ref-9)
10. The Convention on the Rights of the Child, 1989 – Article 7(1). Cf. that everyone has the right to a nationality under the Universal Declaration of Human Rights, 1948 – Article 15, the Convention Relating to the Status of Stateless Persons, 1954, and the Convention on the Reduction of Statelessness, 1961. [↑](#footnote-ref-10)
11. The Convention on the Rights of the Child, 1989 – Article 8(1). [↑](#footnote-ref-11)
12. WHO Guiding Principles on Human Cell, Tissue and Organ Transplantation, 1991, 2010; European Convention on Biomedicine and Human Rights, 1997; EU Charter of Fundamental Rights, 2000; Additional Protocol to the Convention on Human Rights and Biomedicine concerning Transplantation of Organs and Tissues of Human Origin, 2002; WMA Statement on Human Organ Donation and Transplantation, 2000, 2006; Declaration of Istanbul on Organ Trafficking and Transplant Tourism, 2008; Directive 2010/45/EU of the European Parliament and of the Council of 7 July 2010 on standards of quality and safety of human organs intended for transplantation. [↑](#footnote-ref-12)
13. Universal Declaration of Human Rights, 1948 – Article 4. [↑](#footnote-ref-13)
14. League of Nations, Convention to Suppress the Slave Trade and Slavery, 1926; United Nations, Supplementary Convention on the Abolition of Slavery, the Slave Trade, and Institutions and Practices Similar to Slavery, 1956; United Nations Protocol to Prevent, Suppress and Punish Trafficking in Persons, especially Women and Children, 2000; the Council of Europe Convention on Action against Trafficking in Human Beings, 2005; and Directive 2011/36/EU of the European Union on trafficking in human beings [↑](#footnote-ref-14)
15. The Convention on the Elimination of all forms of Discrimination Against Women, 1979 – Article 6; United Nations Protocol to Prevent, Suppress and Punish Trafficking in Persons, especially Women and Children, 2000. [↑](#footnote-ref-15)
16. United Nations Protocol to Prevent, Suppress and Punish Trafficking in Persons, especially Women and Children, 2000; the Optional Protocol to the Convention on the Rights of the Child on the sale of children, child prostitution and child pornography, 2002. See also, the obligation of states to combat the illicit transfer of children abroad, under Article 11 of the Convention on the Rights of the Child, 1989. [↑](#footnote-ref-16)
